# Isolation and characterization of a sequence type 25 carbapenem-resistant hypervirulent *Klebsiella pneumoniae* from the mid-south region of China

**DOI:** 10.1186/s12866-019-1593-5

**Published:** 2019-09-18

**Authors:** Jun Li, Zi-Yan Huang, Ting Yu, Xiao-Yan Tao, Yong-Mei Hu, Hai-Chen Wang, Ming-Xiang Zou

**Affiliations:** Department of Clinical Laboratory, Xiangya Hospital, Central South University, Changsha, 410008 Hunan China

**Keywords:** Carbapenem-resistant, Carbapenemase, Hypervirulent, *Klebsiella pneumoniae*, Serotype

## Abstract

**Background:**

The molecular characterization of carbapenem-resistant hypervirulent *Klebsiella pneumoniae* (CR-hvKP) isolates is not well studied. Our goal was to investigate the molecular epidemiology of CR-hvKP strains that were isolated from a Chinese hospital.

**Results:**

All clinical carbapenem-resistant *K. pneumoniae* (CR-KP) isolates were collected and identified from patient samples between 2014 and 2017 from a Chinese hospital. The samples were subjected to screening for CR-hvKP by string test and the detection of the *aerobactin* gene. CR-hvKP isolates were further confirmed through neutrophil phagocytosis and a mice lethality assay. The CR-hvKP isolates were investigated for their capsular genotyping, virulence gene profiles, and the expression of carbapenemase genes by PCR and DNA sequencing. Multilocus sequence type (MLST) and pulsed-field gel electrophoresis (PFGE) were performed to exclude the homology of these isolates. Twenty strains were identified as CR-hvKP. These strains were resistant to imipenem and several other antibiotics, however, most were susceptible to amikacin. Notably, two isolates were not susceptible to tigecycline. Capsular polysaccharide synthesis genotyping revealed that 17 of the 20 CR-hvKP strains belonged to the K2 serotype, while the others belonged to serotypes other than K1, K2, K5, K20, and K57. The strains were found to be positive for 10 types of virulence genes and a variety of these genes coexisted in the same strain. Two carbapenemase genes were identified: *bla*_KPC-2_ (13/20) and *bla*_NDM-1_ (1/20). PFGE typing revealed eight clusters comprising isolates that belonged to MLST types ST25, ST11 and ST375, respectively. PFGE cluster A was identified as the main cluster, which included 11 isolates that belong to ST25 and mainly from ICU department.

**Conclusions:**

Our findings suggest that hospital-acquired infections may contribute in part to the CR-hvKP strains identified in this study. It also suggests that ST25 CR-hvKP strain has a clonal distribution in our hospital. Therefore, effective surveillance and strict infection control strategies should be implemented to prevent outbreak by CR-hvKP strains in hospitals setting.

## Background

Hypervirulent *Klebsiella pneumoniae* (hvKP) is a variant of *K. pneumoniae*, and was first reported in Taiwan in 1986 [1]. Compared with classic *K. pneumoniae* (cKP), hvKP causes life-threatening, community-acquired infection, especially in younger, healthy global population [2].

In previous studies, the resistance of hvKP to commonly used antimicrobial agents has rarely been reported, except for an intrinsic resistance to ampicillin [3]. However, there has been an increased occurrence of multiple-resistant hvKP strains, including extended spectrum β-lactamases-producing isolates and third-generation cephalosporin-resistant strains [3–5]. Recently, carbapenem-resistant hypervirulent *K. pneumoniae* (CR-hvKP) isolates have also been described in some case reports [6–9]. Furthermore, several studies have shown that *Klebsiella pneumoniae* carbapenemase (KPC)-like genes have the potential for dissemination among hvKP isolates [8, 9]. Carbapenem-resistant *K. pneumoniae* (CR-KP) isolates have the potential to be converted into CR-hvKP through the acquisition of virulence related plasmids, such as *p*LVPK [9, 10]. The confluence of multidrug resistance and enhanced virulence may cause serious outbreaks and public-health problems.

A variety of factors lead to carbapenem resistance, including the production of carbapenemases, decreased expression of outer membrane proteins and overexpression of efflux pumps [11]. In particular, *K. pneumoniae* have acquired carbapenemases, which are enzymes capable of breaking down most β-lactams, including carbapenems, and thus conferring resistance to these drugs. Carbapenemases can be divided into metallo-carbapenemases (zinc-dependent class B) and non-metallo-carbapenemases (zinc-independent classes A, C, and D), according to their dependency on divalent cations for enzyme activation. The primary carbapenemase that was detected in *K. pneumoniae* was of the class A, such as the KPC enzymes [12]. However, there have only been a few reports of KPC production in hvKP isolates [6, 7, 13, 14].

Multilocus sequence type (MLST) has been used as a powerful technique to genotype and characterize bacterial strains. MLST 258 (ST258) is an important CR-KP strain that is responsible for the extensive global spread of KPC-producing *K. pneumoniae*, while ST11, which is closely related to ST258, is a prevalent clone associated with the spread of KPC-producing *K. pneumoniae* in Asia (particularly in China and Taiwan) [15–17]. Additional sequence types of CR-hvKP isolates have also been reported, including ST23, ST11, ST25, ST65, ST86, etc. [18].

Studies have found that the K1 serotype of hvKP is mainly related to ST23, whereas the K2 serotype of hvKP is related to a more diverse number of sequence types, including ST25, ST65, ST66, and ST86 [18–21]. Notably, ST23 hvKP clones of the K1 serotype are associated with pyogenic liver abscesses, whereas ST65 hvKP clones of the K2 serotype are correlated with various invasive infections [21].

Although many studies have reported hvKP infections, especially involving pyogenic liver abscesses, there is limited literature regarding the prevalence, and molecular characteristics of CR-hvKP isolates. In this study, we report 20 CR-hvKP isolates that were identified from a hospital in Hunan, mid-south China. We further investigated the molecular characteristics of these CR-hvKP isolates. Our study provides novel insight into the prevalence of CR-hvKP and may provide important information regarding the treatment and prevention CR-hvKP infections.

## Results

### Determination and clinical characteristics of CR-hvKP

A total of 562 CR-KP isolates were collected. The results of the string test show that 26 CR-KP isolates were positive, with a rate of 4.6%. Twenty-three CR-KP isolates were positive for the *aerobactin* gene. The combination of the results of the string test and the detection of the *aerobactin* gene resulted in 20 CR-hvKP isolates being filtered out (Table 1). The 20 CR-hvKP isolates were from different samples of 15 patients, 13 hospitalized patients and 2 outpatients. All of these patients were males, which is consistent with the findings of a previous study [22]. The main specimens were sputum samples (65.0%, 13/20; Table 1). It is worth noting that seven of the CR-hvKP isolates were detected in two patients (P1 and P3) during multiple hospitalizations. P1 had three consecutive visits to two different wards of the hospital and a total of four CR-hvKP isolates (CS11, CS12, CS70 and CS129) were detected. P3 had two consecutive visits to the same ward of the hospital and three CR-hvKP isolates (CS15, CS48 and CS57) were identified.

**Table 1:** Patient information and clinical features of the CR-hvKP isolates

Strain Number	Patient Number	Age-range	Department	Collection Date	Source	Outcome	Resistance Determinants Genes	PFGE type	MLST
CS1	P2	21–30	ICU	2016/12/27	csf	Death	*bla*_KPC-2_, *bla*_SHV-1_, *bla*_TEM-1_, *bla*_CTX-M-3_	A	25
CS11	P1^a^	41–50	ICU	2016/11/28	sp	Survived	*bla*_KPC-2_, *bla*_SHV-1_, *bla*_TEM-1_, *bla*_CTX-M-3_	A	25
CS12	P1	41–50	ICU	2016/12/6	sp	Survived	*bla*_KPC-2_, *bla*_SHV-1_, *bla*_TEM-1_, *bla*_CTX-M-3_	A	25
CS15	P3^a^	51–60	IMD	2016/12/21	sp	Survived	*bla*_KPC-2_, *bla*_SHV-1_, *bla*_TEM-1_, *bla*_CTX-M-3_	A	25
CS17	P4	71–80	ICU	2016/12/3	sp	Giving up Treatment^b^	*bla*_KPC-2_, *bla*_SHV-1_, *bla*_TEM-1_, *bla*_CTX-M-3_	A	25
CS45	P5	41–50	ICU	2016/11/15	sp	Giving up treatment	*bla*_KPC-2_, *bla*_TEM-1_, *bla*_CTX-M-3_	A	25
CS47	P6	71–80	ICU	2016/12/28	blood	Giving up treatment	*bla*_KPC-2_, *bla*_TEM-1_, *bla*_CTX-M-3_	A	25
CS48	P3	51–60	IMD	2016/12/31	sp	Survived	*bla*_KPC-2_, *bla*_SHV-1_, *bla*_TEM-1_, *bla*_CTX-M-3_	A	25
CS57	P3	51–60	IMD	2017/3/11	sp	Survived	*bla*_KPC-2_, *bla*_SHV-1_, *bla*_TEM-1_, *bla*_CTX-M-3_	A	25
CS60	P14	61–70	CSD	2017/6/16	puncture fluid	Giving up treatment	*bla*_SHV-1_	H	25
CS61	P15	31–40	ICU	2017/6/4	drainage liquid	Death	*bla*_SHV-1_	H	25
CS62	P8	51–60	ICU	2017/6/16	sp	Survived	*bla*_SHV-1_	C	25
CS70	P1	41–50	RD	2017/6/22	sp	Survived	*bla*_TEM-1_, *bla*_CTX-M-3_	A	25
CS80	P13	61–70	Burn Unit	2017/7/2	traumatic secretion	Death	*bla*_KPC-2_, *bla*_SHV-1_, *bla*_TEM-1_, *bla*_CTX-M-65_	G	11
CS90	P9	51–60	Outpatient	2017/3/27	drainage liquid	Survived	*bla*_SHV-1_, *bla*_CTX-M-65_	D	375
CS103	P11	41–50	ICU	2017/5/22	sp	Giving up treatment	*bla*_KPC-2_, *bla*_SHV-1_	F	11
CS118	P12	71–80	ICU	2017/3/18	sp	Giving up treatment	*bla*_KPC-2_, *bla*_SHV-1_, *bla*_TEM-1_, *bla*_CTX-M-65_	F	11
CS127	P10	41–50	Outpatient	2017/5/11	blood	Survived	*bla*_SHV-1_	E	25
CS129	P1	41–50	ICU	2017/3/16	sp	Survived	*bla*_KPC-2_, *bla*_SHV-1_, *bla*_CTX-M-3_	A	25
CS59497	P7	51–60	ICU	2014/10/5	sp	Survived	*bla*_NDM-1_, *bla*_SHV-1_, *bla*_TEM-1_	B	25

Department: indicates the department at the Xiangya Hospital, where the samples were collected; *ICU* Intensive care unitm, *IMD* Integrative Medicine Department, *CSD* Cerebrovascular Surgery Department, *RD* Rehabilitation Department, *mCIM* Modified Carbapenem Inactivation Method, *PFGE* Pulsed Field Gel Electrophoresis, *MLST* Multilocus sequence type

“*CSF*” Cerebrospinal fluid, “*Sp*” Sputum

^a^Patients P1 and P3 are immunocompromised patients. Repeated infections occurred in these two patients during 2016 and 2017

^b^The patient’s condition deteriorated so the patient stopped any further treatment

The 20 CR-hvKP isolates had high drug resistance rates for most of the antibacterial drugs that were tested, except for tigecycline and amikacin. Two isolates were not susceptible to tigecycline (CS45 and CS129, with MICs of 1.5 μg/mL and 4 μg/mL, respectively) (shown in Table 2).

**Table 2:** Resistance to antimicrobial agents of the CR-hvKP isolates

Antimicrobial Agents	R (%)	I (%)	S (%)
Ceftazidime	65.0	15.0	20.0
Ceftriaxone	85.0	0.0	15.0
Cefepime	45.0	30.0	25.0
Aztreonam	80.0	0.0	20.0
Piperacillin/Tazobactam	60.0	20.0	20.0
Amikacin	15.0	0.0	85.0
Gentamycin	55.0	5.0	40.0
Ciprofloxacin	80.0	0.0	20.0
Levofloxacin	45.0	5.0	50.0
Trimethoprim-Sulfamethoxazole	65.0	0.0	35.0
Imipenem	100.0	0.0	0.0
Tigecycline	5.0	5.0	90.0

*R* Resistant, *I* Intermediary, *S* Susceptible

**Fig. 1:**
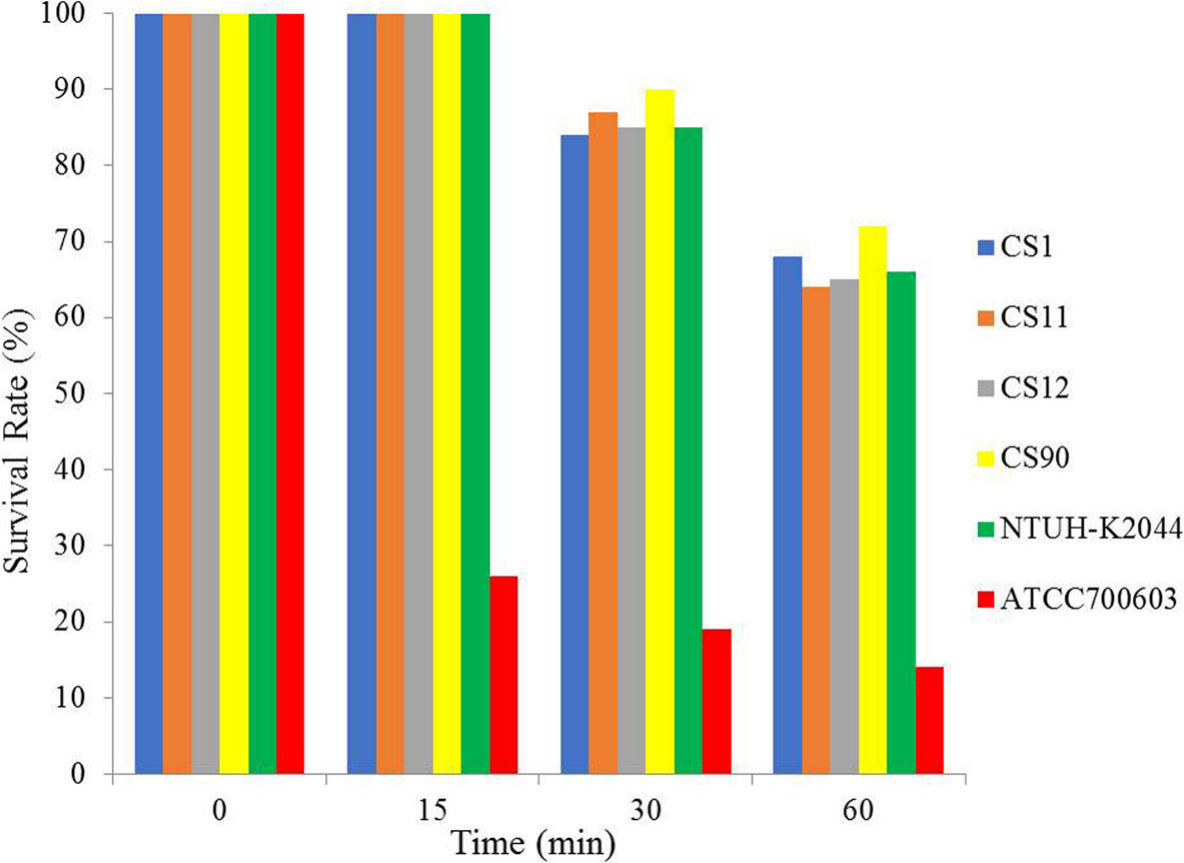
Neutrophil phagocytosis analysis of four randomly selected CR-hvKP isolates (CS1, CS11, CS12 and CS90). NTUH-K2044: a hypervirulent wild strain of *K. pneumoniae*; ATCC 700603: a standard strain of *K. pneumoniae* with low virulence and low resistance

**Fig. 2:**
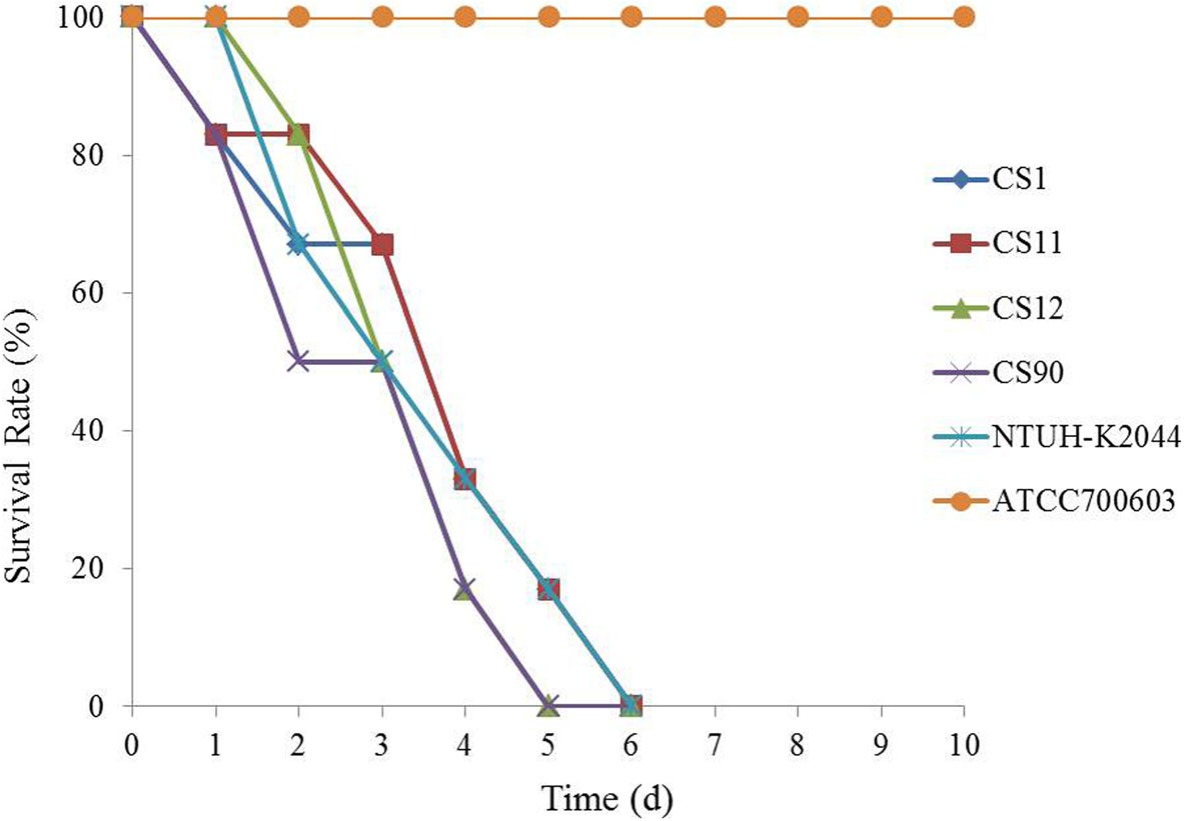
Mice lethality assay using four randomly selected CR-hvKP isolates (CS1, CS11, CS12 and CS90). NTUH-K2044: a hypervirulent wild strain of *K. pneumoniae*; ATCC 700603: a standard strain of *K. pneumoniae* with low virulence and low resistance

Four CR-hvKP isolates were randomly selected for neutrophil phagocytosis and mice lethality assay. The results of the neutrophil phagocytosis show that the survival rates of the four tested CR-hvKP strains and NTUH-K2044 (a hypervirulent wild strain) were significantly higher than that of *K. pneumoniae* ATCC 700603 (a standard strain for low virulence and low resistance; *P* < 0.05; Fig. 1). The mice lethality assay results show that the survival time of mice that were infected with the CR-hvKP isolates or NTUH-K2044 was 5–6 d, while those infected with *K. pneumoniae* ATCC 700603 all survived the monitoring period (10 d; Fig. 2).

### Capsular serotyping and virulence genes

Among the 20 CR-hvKP isolates, 17 were identified as the K2 serotype, and 3 (CS80, CS103, and CS118) were serotypes other than K1, K2, K5, K20, and K57 (Table 3). We also found that isolates were positive for each of the 10 virulence genes tested, but the proportion of isolates positive for each virulence gene varied, from 10 to 100% (Table 3). Some virulence genes had a low positive rate, such as *allS* (10%) and *fimH* (30%). It suggests that these genes might be less important for high virulence in these strains.

**Table 3:** Serotypes and the presence of virulence genes in the CR-hvKP isolates

Strain Number	Serotyping	Virulence Gene									
		*rmpA*	*iucB*	*iroB*	*uge*	*wabG*	*ureA*	*allS*	*ybtS*	*fimH*	*entB*
CS1	K2	–	+	+	+	+	+	–	+	+	+
CS11	K2	+	+	+	+	+	+	–	+	–	+
CS12	K2	+	+	+	+	+	+	–	+	+	+
CS15	K2	+	+	+	+	+	+	–	+	+	+
CS17	K2	+	+	+	+	+	+	–	–	–	+
CS45	K2	+	+	+	+	+	+	–	–	–	+
CS47	K2	+	+	+	+	+	+	–	–	–	+
CS48	K2	+	+	+	+	+	+	–	+	–	+
CS57	K2	+	+	+	+	+	+	–	+	–	+
CS60	K2	+	+	–	+	+	+	+	–	–	+
CS61	K2	+	+	–	+	+	+	+	–	–	+
CS62	K2	+	–	+	+	+	+	–	–	–	+
CS70	K2	+	–	+	+	+	+	–	–	–	+
CS80	NA	–	+	–	+	+	+	–	+	+	+
CS90	K2	+	+	–	+	+	+	–	–	–	+
CS103	NA	–	+	–	+	+	+	–	+	+	+
CS118	NA	–	+	–	+	+	+	–	–	–	+
CS127	K2	+	+	–	+	+	+	–	–	–	+
CS129	K2	+	+	+	+	+	+	–	–	+	+
CS59497	K2	+	+	+	+	+	+	–	+	–	–
Positive rate of genes (%)		80	90	65	100	100	100	10	45	30	95

### Carbapenemase phenotype and carbapenemase gene detection

To further confirm the presence of carbapenemase, carbapenemase genes were tested and two families of carbapenemase genes were identified: *bla*_KPC-2_ (13/20) and *bla*_NDM-1_ (1/20; Table 1). All strains were found to be negative for the other carbapenemase genes tested, including *bla*_VIM_, *bla*_IMP_ and *bla*_OXA-48_. As shown in Table 1, two isolates (CS80 and CS103) that were negative for mCIM were *bla*_KPC_-positive. In addition, other β-lactamase genes, including *bla*_TEM-1_, *bla*_SHV-1_ and *bla*_CTX-M-3_, were frequently detected in these CR-hvKP isolates. Notably, three stains carried *bla*_CTX-M-65_ (Table 1).

### CR-hvKP fingerprinting

**Fig. 3:**
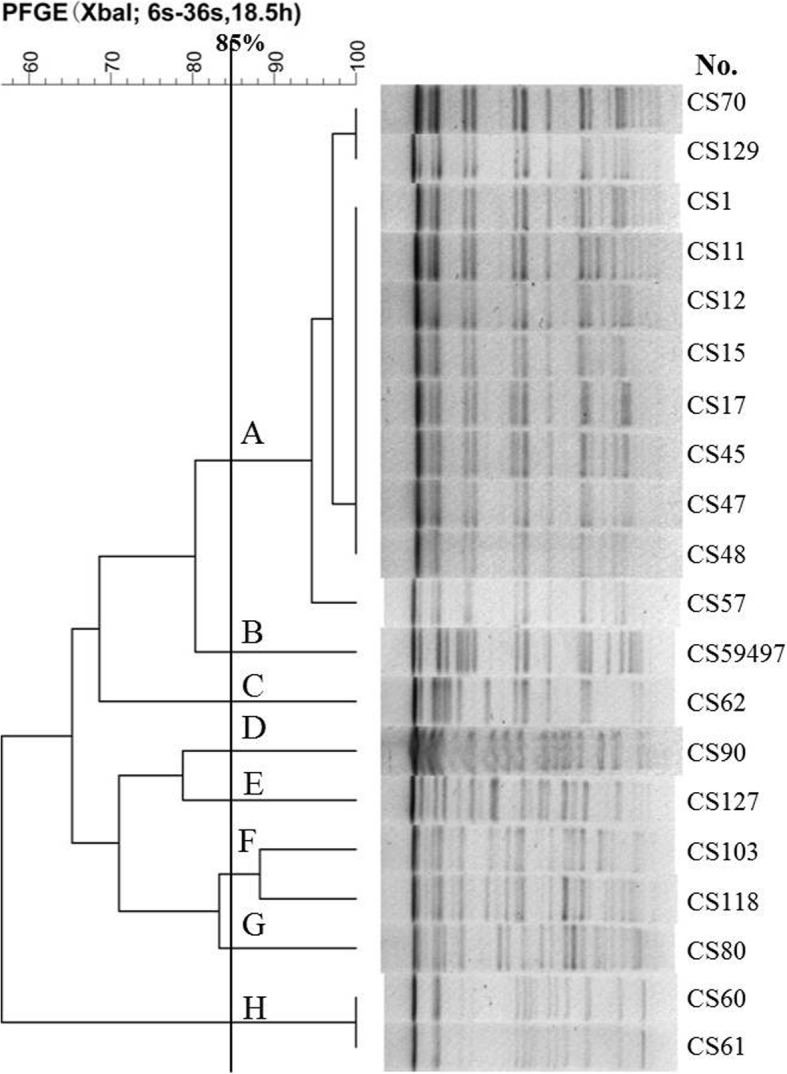
Relationships of the 20 CR-hvKP isolates based on Pulsed Field Gel Electrophoresis (PFGE). The 20 CR-hvKP isolates were analyzed by PFGE using *XbaI*. The interpretation of the PFGE patterns was performed with BioNumerics software using the Dice similarity coefficient. The tree indicates relative genetic similarity and was constructed on the basis of the unweighted pair group method of averages (UPGMA). A PFGE pattern with more than 85% DNA bands that are different from the others is taken to be a unique PFGE pattern

PFGE analysis revealed that the 20 CR-hvKP isolates showed 8 PFGE types (type A to type H), with type A as the dominating type, being expressed in 55.0% of the isolates (11/20). The majority of the type A isolates came from the intensive care unit (ICU; Fig. 3; Table 1). MLST detected three sequence types, including ST25, ST11 and ST375. ST25 was the main sequence type, accounting for 80.0% of the isolates. Among them, PFGE types A, B, C, E and H belong to ST25, type F and type G are ST11, while type D belongs to ST375 (Table 1). Interestingly, three isolates (CS11, CS12 and CS129) were all from samples that were collected from patients in the ICU that were collected during the same time period, and had the same carbapenemase phenotypes and PFGE profile, suggesting that they may be hospital-acquired infections.

## Discussion

Increasing rates of hvKP infection have been reported worldwide over the past few decades. However, until the last several years, only a few cases of CR-hvKP infections have been reported [2, 6–9]. In this study, 20 CR-hvKP isolates were identified from a single hospital in mid-south China. Several of the isolates were identified from the same patients. Additionally, several of these isolates were sampled from the ICU and share some common phenotypes, suggesting that they might be a hospital-acquired infection. Notably, hvKP is considered to be the predominant cause of pyogenic liver abscess [18, 23, 24]. However, we found that the isolates were mostly identified from sputum samples taken from patients that did not have symptoms of a pyogenic liver abscess, suggesting that CR-hvKP can also cause infection or colonize in other parts of the human body.

Serotypes K1 and K2 are the most common and virulent strains of hvKP [25, 26]. Similarly, in this study we found 17 isolates that belonged to serotype K2 and the remaining 3 were nontypable. Serotype K2 has been identified in many countries, including China, Germany, Spain and Singapore [10, 19, 24, 27, 28]. Serotype K2 generally belongs to a diverse sequence of types, such as ST25, ST65, ST66 and ST86 [18]. In our current study, we found that the serotype K2 CR-hvKP isolates all belonged to ST25, except for one CR-hvKP strain (ST375).

mCIM is a novel method that was recommended by the CLSI in 2017 for the detection of carbapenemase phenotypes and is considered to have high sensitivity and specificity [29, 30]. Our results show that 60% (12/20) of the CR-hvKP strains were mCIM positive. We then tested the strains for carbapenemase genes and 14 of the 20 CR-hvKP strains were positive. All of the 12 mCIM-positive strains were positive for the carbapenemase gene, indicating that the specificity of mCIM is as high as 100.0%. We also found that 2 mCIM-negative CR-hvKP strains were positive for the *bla*_KPC-2_ gene. We previously investigated 259 clinical *K. pneumoniae* strains [75 CR-KP and 184 carbapenem-susceptible *K. pneumoniae* (CS-KP)] by mCIM and PCR assay. Our results showed that 69 of the tested 75 CR-KP strains were mCIM positive, in accordance with the results of PCR assay, while all of the CS-KP isolates were mCIM negative. Among the six negative CR-KP isolates, three were positive for carbapenem genes (*bla*_KPC_/*bla*_VIM_) with a high expression of AmpC (Jun Li, Ziyan Huang, Xiaoyan Tao, etc., unpublished data). We speculate that the carbapenem gene may be partially deleted. For example, a truncated transposon Tn*4401* variant Tn*4401d* is not able to express the *bla*_KPC_ gene, causing a loss of carbapenemase activity, subsequently leading to the negative results of mCIM [31].

The epidemiology of KPC-producing *K. pneumoniae* has been mainly associated with the global expansion of cKP resistance to carbapenems. However, there are limited reports of CR-hvKP strains that carry the *bla*_KPC_ gene [6, 7, 13, 14]. In this study, we found that the KPC-producing CR-hvKP strains were predominant, suggesting that KPC was the main mechanism of carbapenem resistance in hvKP strains. In addition, it has been reported that the *bla*_KPC_ gene can be transferred to hvKP by plasmid conjugation [10], suggesting that drug resistance genes can be acquired by hvKP from the environment, producing a hypervirulent strain with high drug resistance. Previous studies showed that most of KPC-producing *K. pneumoniae* isolates belonged to ST11, which is a single-locus variant of the pandemic ST258 clone and is considered to be a high-risk epidemic clone owing to its ability to spread rapidly and disseminate carbapenemases. Several studies also found a few KPC-producing *K. pneumoniae* isolates belonging to ST11 and clone dissemination in hospitals. For example, one report showed that five patients died of severe pneumonia in a hospital due to infection of KPC-producing hvKP isolates belonging to ST11 [9]. In our study, 3 patients were infected with KPC-producing hvKP isolates belonging to ST11 in the hospital and one died. It was rarely reported that KPC-producing hvKP isolates belong to ST25 [27]. However, we found 10 KPC-producing hvKP isolates all belonging to ST25, suggesting that ST25 may play an important role in promoting the dissemination of *bla*_KPC_-carried hvKP isolates in our hospital. It is worth noting that one CR-hvKP isolate was positive for the *bla*_NDM-1_ gene, which was first identified in 2010 in a extensively drug-resistant (XDR) *K. pneumoniae* clinical isolate [32].

PFGE is a molecular typing technique that is broadly used in bacterial epidemiological studies. PFGE analysis of the 20 CR-hvKP strains identified eight genotypes from type A to type H. Type A was the main genotype, accounting for 55.0% of the strains. Interestingly, this genotype was mainly isolated from patients in the ICU, suggesting that the CR-hvKP strains that were isolated from this hospital could possibly have clone transmission.

## Conclusions

In conclusion, our study revealed that a small percentage (20 out of 562) CR-KP are hypervirulent and that these CR-hvKP strains are highly resistant to most of the commonly used antibacterial drugs, which could bring great challenges to clinical anti-infection treatment. We believe that our findings may provide new insights into the treatment and prevention of CR-hvKP infections, especially in China. One limitation of our current study is the possible inconsistency of sample collection, due to the difficulty and limitation of working in a clinical setting. A more comprehensive and consistent sample collection (such as collection from the same sample source) could greatly improve the outcome.

## Methods

### Ethics statement and study subjects

This study was carried out in accordance with the recommendations of the Ethics Committee of Central South University (Changsha, Hunan Province, China) and with the 1964 Helsinki declaration and its later amendments or comparable ethical standards. The protocol was approved by the Ethics Committee of Central South University (Changsha, Hunan Province, China). All of the participants provided written consent prior to the study.

### Collection, identification and antimicrobial susceptibility testing of *K. pneumoniae* clinical isolates

*K. pneumoniae* isolates, which were identified by Matrix Assisted Laser Desorption/Ionization Time of Flight Mass Spectrometry (MALDI-TOF MS; Bruker Daltonics GmbH, Germany) and examined by VITEK-2 automated microbiology analyzer (bioMérieux, Marcy l’Etoile, France), were collected from patients at the Xiangya Hospital Central South University between September 1st 2014 and September 31st 2017. This hospital has 3500 beds and is located in Changsha, Hunan Province, China. To avoid duplicate samples, the majority of isolates were collected from different types of specimen from each patient. In some cases, isolates were collected from the same type of specimen. In these cases, the interval between the collection of the two samples was at least 1 week. *Escherichia coli* ATCC 25922 and *K. pneumoniae* ATCC 700603 (American Type Culture Collection, Manassas, VA) were used as controls for the species identification.

The minimal inhibitory concentrations (MICs) of imipenem for imipenem-resistant *K. pneumoniae* were verified by E-test method according to the guidelines recommended by the Clinical and Laboratory Standards Institute [29]. The MICs of tigecycline were performed by E-test method and interpretation of the results was based on break points of the European Committee on Antimicrobial Susceptibility Testing (EUCAST) (http://www.eucast.org/clinical_breakpoints). *E. coli* ATCC 25922 was used as a control.

### Identification of CR-hvKP isolates

To identify CR-hvKP isolates, the CR-KP isolates were screened by string test and the detection of the *aerobactin* gene, as has been described previously (Zhang et al., 2016b). The primers used for PCR were as follows: *aerobactin* forward, 5′-GCATAGGCGGATACGAACAT-3′; *aerobactin* reverse, 5′-CACAGGGCAATTGCTTACCT-3′. The reaction mixture was kept at 95 °C for 5 min, followed by 30 cycles of 95 °C for 1 min, 50 °C for 1 min, 72 °C for 1 min, and 72 °C for 10 min. The PCR products were visualized and analyzed by agarose gel electrophoresis and sequencing.

### Neutrophil phagocytosis

Neutrophil phagocytosis was performed as described previously [6, 19]. Briefly, four CR-hvKP isolates were selected for analysis. The 4 CR-hvKP isolates include all three sequence types (2 ST25 and 1 ST11 were randomly selected and 1 ST375 was selected) (Table 1). *K. pneumoniae* ATCC 700603 (a standard strain for low virulence and low resistance) and *K. pneumoniae* NTUH-K2044 (a hypervirulent wild strain) were used as the controls. Neutrophil cells were isolated from the venous blood of healthy volunteers. A solution of 200 μL 1 × 10^6^ neutrophil cells (or 200 μL sterilized saline in the control group), 200 μL 1 × 10^6^ CR-hvKP suspension and 600 μL RPMI 1640 medium (Biosun, Shanghai, China) was mixed in a cell culture plate, and incubated on a rocking bed at 37 °C with constant shaking. Ten microliters of 0.1% saponin were added to each plate at 0, 15, 30 and 60 min incubation time. The plates were then placed on ice for 15 min and the CR-hvKP isolates were inoculated on Luria-Bertani solid medium overnight. The colony formation unit (CFU) was counted. The survival rate was determined using the following equation: (CFU in the presence of neutrophils)/(CFU with sterilized saline) × 100. The experiment was repeated three times.

### Mouse lethality assay

The mouse lethality assay was performed as described previously [6, 19]. Briefly, four CR-hvKP isolates were randomly selected for the experiment and *K. pneumoniae* ATCC 700603 and *K. pneumoniae* NTUH-K2044 were used as the controls. CR-hvKP suspensions of 1 × 10^6^ cfu/mL were injected into the abdominal cavity of BALB/C mice (that were obtained from the Animal Center of the Central South University). The mortality rates were observed over 10 days. Three mice were used in each CR-hvKP isolate condition and the experiment was repeated three times. After the experiments, mice were all euthanized using 100% CO_2_ at a flow rate of ~ 2 l per minute.

### Modified carbapenem inactivation method (mCIM)

To identify the carbapenemase phenotypes, mCIM was performed with meropenem for all of the CR-hvKP isolates, according to the operation standard of CLSI [29]. The zone diameter around the meropenem was measured. The result was considered to be positive when the diameter was < 16 mm, while the result was considered to be negative when the diameter was > 19 mm. The result was considered to be intermediary when the inhibition zone diameter was between 16 and 18 mm. *K. pneumoniae* ATCC BAA-1705 and *K. pneumoniae* ATCC BAA-1706 were used as the positive and negative controls, respectively [29].

### Capsular serotyping, detection of resistance genes and virulence genes

PCR was used to detect the serotype genes (including K1, K2, K5, K20, and K57), virulence genes (*rmpA*, *iucB*, *ironB*, *uge*, *wabG*, *ureA*, *alls*, *ybtS*, *fimH*, and *entB*), carbapenemase genes (*bla*_KPC_, *bla*_NDM_, *bla*_VIM_, *bla*_IMP_, and *bla*_OXA-48_), and other β-lactamase genes (*bla*_CTX − M_, *bla*_TEM_, *bla*_SHV_, *bla*_DHA_, and *bla*_CMY_). PCR primers and conditions have been described elsewhere [33, 34]. All the positive products were sequenced and analyzed using the BLAST website (https://blast.ncbi.nlm.nih.gov/blast.cgi).

### Molecular epidemiology

Genetic relatedness among the CR-hvKP isolates was determined by pulsed-field gel electrophoresis (PFGE). In brief, genomic DNA was prepared by embedding *K. pneumoniae* cells in agarose plugs (ThermoFisher, China), followed by *Xba*I (Promega, USA) digestion for 18.5 h at 37 °C. Electrophoresis was performed at 14 °C for 20 h using the Bio-Rad CHEF III system (120° angle, 6 V/cm, switch times of 6 and 36 s). Cluster analysis was performed with BioNumerics software Version 5.1 (Applied Maths, Austin, TX) using the Dice Similarity Coefficient. Isolates with pattern similarities > 85% were considered to be from the same PFGE cluster [35]. A subset of isolates that represented the different PFGE clusters were further studied by multilocus sequence typing (MLST), according to the Institut Pasteur scheme [36].

### Statistical analysis

Statistical analysis was conducted using SPSS19.0 software (SPSS Inc., USA). For the mouse modality test, survival curves were assessed using Kaplan-Meier analysis and the log-rank test. A value of *P* < 0.05 was considered to be statistically significant.

## Data Availability

The datasets generated and analyzed during the current study are available from the corresponding author on reasonable request.

## Abbreviations

BLAST: Basic Local Alignment Search Tool
CFU: Colony Formation Unit
cKP: classic *K. pneumoniae*
CLSI: Clinical and LaboratoryStandards Institute
CR-hvKP: Carbapenem-resistant hypervirulent *Klebsiella pneumoniae*
CR-KP: Carbapenem-resistant *Klebsiella pneumoniae*
EUCAST: European Committee on Antimicrobial Susceptibility Testing
hvKP: hypervirulent *Klebsiella pneumoniae*
*K. pneumoniae*: *Klebsiella pneumoniae*
KPC: *Klebsiella pneumoniae* carbapenemase
mCIM: modified Carbapenem Inactivation Method
MICs: Minimal Inhibitory Concentrations
MLST: Multilocus sequence type
PCR: Polymerase Chain Reaction
PFGE: Pulsed-field gel electrophoresis
SPSS: Statistical Package for the Social Sciences
ST: Sequence Type
XDR: Extensively Drug-resistant
